# Evaluation of the Dimensional Accuracy of Robot-Guided Laser Osteotomy in Reconstruction with Patient-Specific Implants—An Accuracy Study of Digital High-Tech Procedures

**DOI:** 10.3390/jcm13123594

**Published:** 2024-06-19

**Authors:** Bilal Msallem, Lara Veronesi, Michel Beyer, Florian S. Halbeisen, Michaela Maintz, Adrian Franke, Paula Korn, Adrian Dragu, Florian M. Thieringer

**Affiliations:** 1 University Center for Orthopedics, Trauma and Plastic Surgery, Medical Faculty and University Hospital Carl Gustav Carus, Technische Universität Dresden, DE-01307 Dresden, Germany; adrian.dragu@ukdd.de; 2 Medical Additive Manufacturing Research Group (Swiss MAM), Department of Biomedical Engineering, University of Basel, CH-4123 Allschwil, Switzerland; lara.veronesi@usb.ch (L.V.); michel.beyer@usb.ch (M.B.); michaela.maintz@usb.ch (M.M.); florian.thieringer@usb.ch (F.M.T.); 3 Clinic of Oral and Cranio-Maxillofacial Surgery, University Hospital Basel, CH-4031 Basel, Switzerland; 4 Surgical Outcome Research Center, Department of Clinical Research, University of Basel c/o University Hospital of Basel, CH-4001 Basel, Switzerland; floriansamuel.halbeisen@usb.ch; 5 Institute for Medical Engineering and Medical Informatics, University of Applied Sciences and Arts Northwestern Switzerland, CH-4132 Muttenz, Switzerland; 6 Department of Oral and Maxillofacial Surgery, Medical Faculty and University Hospital Carl Gustav Carus, Technische Universität Dresden, DE-01307 Dresden, Germany; adrian.franke@ukdd.de (A.F.); paula.korn@ukdd.de (P.K.)

**Keywords:** 3D printing, dimensional measurement accuracy, laser ablation, mandibular osteotomy, mandibular reconstruction, precision medicine, robotic surgical procedures

## Abstract

**Background/Objective**: With the rapid advancement in surgical technologies, new workflows for mandibular reconstruction are constantly being evaluated. Cutting guides are extensively employed for defining osteotomy planes but are prone to errors during fabrication and positioning. A virtually defined osteotomy plane and drilling holes in robotic surgery minimize potential sources of error and yield highly accurate outcomes. **Methods**: Ten mandibular replicas were evaluated after cutting-guided saw osteotomy and robot-guided laser osteotomy following reconstruction with patient-specific implants. The descriptive data analysis summarizes the mean, standard deviation (SD), median, minimum, maximum, and root mean square (RMS) values of the surface comparison for 3D printed models regarding trueness and precision. **Results**: The saw group had a median trueness RMS value of 2.0 mm (SD ± 1.7) and a precision of 1.6 mm (SD ± 1.4). The laser group had a median trueness RMS value of 1.2 mm (SD ± 1.1) and an equal precision of 1.6 mm (SD ± 1.4). These results indicate that robot-guided laser osteotomies have a comparable accuracy to cutting-guided saw osteotomies, even though there was a lack of statistical significance. **Conclusions**: Despite the limited sample size, this digital high-tech procedure has been shown to be potentially equivalent to the conventional osteotomy method. Robotic surgery and laser osteotomy offers enormous advantages, as they enable the seamless integration of precise virtual preoperative planning and exact execution in the human body, eliminating the need for surgical guides in the future.

## 1. Introduction

Cutting bones is an everyday necessity in many surgical disciplines. There are various tools for performing osteotomies, e.g., mallets, chisels, and rotating as well as oscillating instruments. Although these instruments have been used for decades and have been tried and tested in daily use, some relevant disadvantages still need to be eliminated [1,2,3]. For example, the process of cutting bone by sawing can generate vibration and heat. This can lead to bone chipping and the clogging of exposed bone surfaces, necrosis, as well as impaired bone healing. In addition, there are limitations to the possible cutting geometry, implying that not every cutting shape can be attained with conventional osteotomy tools [4,5,6,7,8]. As a result, surgical methods and procedures are constantly evolving. A growing number of studies are investigating the use of robotic surgery in hard tissue cutting, which can remove many of the geometric constraints that currently exist and reduce the potential for inaccuracies due to human error. Furthermore, the use of robot-guided osteotomy planning with geometric cuts can result in greater stability and a larger bone contact surface, which can facilitate the healing of the reconstructed bone [9]. Digital workflows are increasingly being implemented in surgical processes, particularly in reconstructive surgery. The benefits of digital workflows encompass precise preoperative virtual planning, the early identification of potential intraoperative challenges, the feasibility of prefabrication, and the saving of time in the operating room [10]. Accurate preoperative planning minimizes surgeon-dependent imprecision and enables superior predictions about the anticipated clinical outcome in hard and soft tissue [10,11,12,13]. This can lead to reduced surgical complication rates and better clinical outcomes [14,15].

Currently, virtual surgical planning and 3D printing are not only used for visualization and preoperative planning, but also for the fabrication of patient-specific cutting guides and, more recently, patient-specific implants. Thus, the entire surgical workflow can be improved by prefabricated cutting guides. However, inaccuracies based on the handling and positioning of the cutting guide may still occur, as the osteotomy is still performed manually [11,16]. Implementing and repeating a preoperatively planned patient-specific cutting path without cutting guides offers considerable advantages. Integrating robot guidance would prove beneficial in this scenario and may also result in the complete exploitation of the precise cutting line when paired with a laser. Furthermore, this approach also holds many biological benefits at the cellular level [11,12,16]. A laser osteotome has an even smaller cutting width, resulting in less bone loss than oscillating instruments [17]. Previous studies have demonstrated a high accuracy when comparing the planned cutting lines with the final cuts conducted by a robot-guided laser [18]. Moreover, there are minimal restrictions on the design of the object’s cutting geometry, as a wide variety of shapes can be produced with great accuracy [19,20,21]. For example, it is possible to create self-stabilizing complex cutting geometries, such as functional cuts, to interlock the intersections in Le Fort I osteotomies [22]. Additional possibilities exist regarding the potential of planning and executing defect reconstructions using free fibula flaps. The application of robot-guided osteotomies in harvesting fibula flaps presents a promising prospect for achieving seamless integration within the surgical procedure [14]. A robotic arm with a navigation system offers numerous advantages in the field of bone cutting, as well as great potential in the three-dimensional repositioning of bones intraoperatively in complex reconstructions [23].

When considering the biological effects of lasers available for osteotomies, Erbium-doped Yttrium Aluminum Garnet (Er:YAG) lasers have shown the most favorable results regarding bone impact. Neodymium–doped Yttrium Aluminum Garnet (Nd:YAG) lasers, on the other hand, are associated with adverse thermal effects, while carbon-dioxide (CO_2_) lasers are associated with bone sequestration, coagulation, and carbonization of the tissue [15,17], whereas the Er:YAG laser works below the critical bone temperature so that no carbonization occurs. With its wavelength of 2940 nm, which is the wavelength absorbed by water and used to cut hydroxyapatite, the laser is indicated where the cutting of hard mineralized tissue is required [1,15,24,25,26]. The Er:YAG laser induces a photothermic effect, resulting in photoablation; specifically, the water content of the tissue absorbs the laser energy, causing a corresponding increase in pressure that leads to microexplosions. These characteristics effectively reduce heat production and minimize carbonization [27,28]. Laser osteotomy preserves the trabecular architecture and pores of the bone, resulting in cutting margins with biologically open cut surfaces and minimized mechanical and thermal damage. This leads to less carbonization of the bone and potentially better bone healing. Laser osteotomes work without mechanical force and represent a non-contact, blood-, heat-, and vibration-reduced alternative to conventional osteotomy methods [7,19,21,22].

So, this study aims to evaluate the accuracy of a robot-guided laser osteotome in terms of outcome after the reconstruction, utilizing patient-specific implants. The findings will offer significant information for the progression and enhancement of laser osteotomies, while affirming the current state of technology and feasible prospects.

## 2. Materials and Methods

### 2.1. Study Protocol

**Figure 1:**
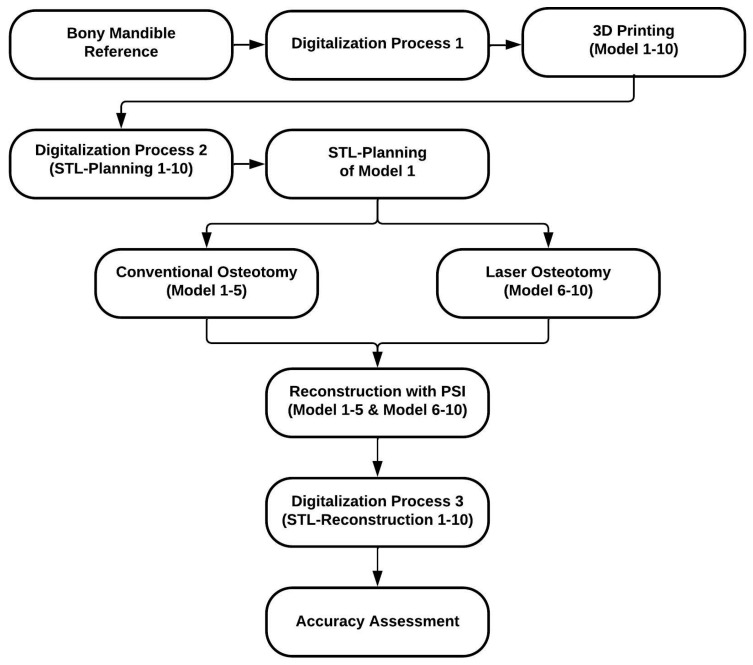
Flowchart of the study protocol.

The study protocol outlines the following procedures (Figure 1) and chronologically presents the study’s methods. The replicas of Models 1–10 mentioned in the flowchart below are the original 3D printed models that were used to cut and reconstruct, while the standard tessellation language (STL) files STL 1–10 refer to the digital datasets that are used for the accuracy assessment. Ethical approval was not required as this study does not report on or involve the use of animal or human data or tissues.

### 2.2. CARLO^®^

**Figure 2:**
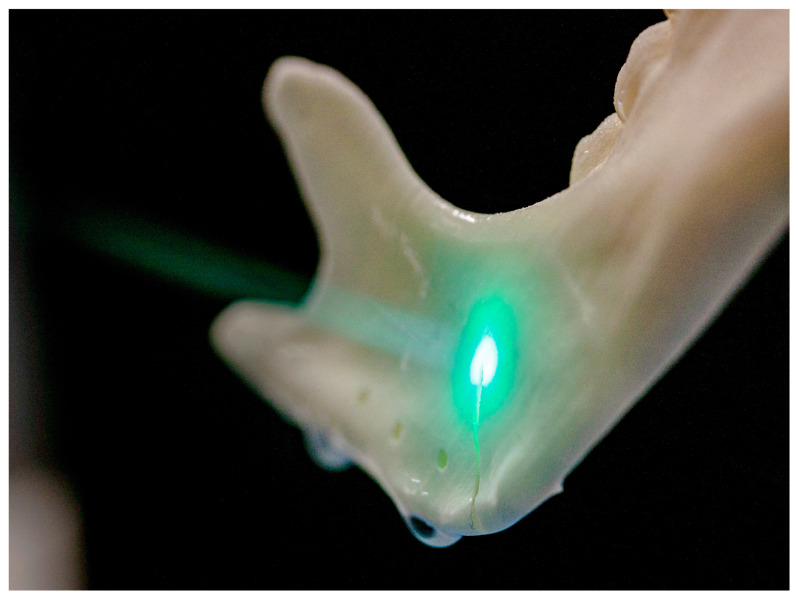
Osteotomy conducted by the laser osteotome.

CARLO^®^ is an acronym for Cold Ablation Robot-guided Laser Osteotome, denoting a robot-guided Er:YAG laser device for thermal ablation. This laser is attached at the distal end of a robotic arm. An aiming beam, represented by a green laser, facilitates visualization prior to and during the cutting process, as depicted below (Figure 2). Further, it also holds a camera and a scanning laser. The maximum cutting depth is 20 mm, with a total width of 1.5 mm. A water spray is constantly applied to cool and rinse the cutting area. A CARLO^®^ procedure pack (AOT AG, Basel, Switzerland) with the necessary disposable materials, such as water tubes for the cooling system, is required for each surgical intervention. In this study, we used the same procedure pack for all osteotomies. We applied the CARLO^®^ primo+ software v. 2.0.x (AOT AG, Basel, Switzerland), which is indicated for laser osteotomies in the mid-face, mandible, fibula, or ex corpore procedures. The cutting can either be planned using preoperative data, such as a computed tomography (CT) scan, or ad hoc adjustments can be made before and during the procedure using the touchscreen interface on the CARLO^®^ trolley.

### 2.3. Fabrication Process of the Mandibular Models

#### 2.3.1. Digitalization Process 1 (Reference Model)

In this study, we digitized an ex vivo bony mandible by scanning it with a white-light desktop optical 3D scanner (EinScan-SP, SHINING 3D Tech. Co., Ltd., Hangzhou, China), using the EinScan-S series software v. 2.7.0.6. We scanned the bony mandible from four different angles (upside, upside down, right side up, and left side up). These four scans are merged to create a digital model exported to an STL file, which served as the basis for the 3D prints of the mandibular models.

#### 2.3.2. 3D Printing of the Models (Replicas) and Digitalization Process 2

The STL file from the digitized bony mandible is exported to the 3D printer’s corresponding software. Based on this STL file, ten mandibular models are printed using selective laser sintering (SLS) technology with a 3D printer EOSINT P 385 (EOS GmbH, Krailling, Germany). The models are made from white polyamide 12 PA 2200 powder (EOS GmbH, Krailling, Germany). The ten printed SLS-mandibular models are scanned using the white-light desktop optical 3D scanner, following the same procedure as the reference model. This way, ten additional STL files labeled STL-Planning 1–10 are generated. 

### 2.4. Conventional Osteotomy by Saw

#### 2.4.1. Osteotomy Design and Cutting Guide Planning

The accuracy of the ten generated STL-Planning files was then compared. As the models exhibited no relevant aberrances, our planning of the osteotomy lines and guides for the conventional osteotomy was grounded in the STL-Planning file of Model 1, using Geomagic Freeform (3D Systems Inc., Rock Hill, SC, USA). The extent of the segmentectomy was defined and virtually marked. We then adjusted the cutting guides to fit the mandible and included guiding tubes for the drilling holes. One guide was used for the osteotomy on the right ramus, whereas the other one was applied to the frontal canine region. Then, we 3D printed the guides from dark gray polyamide 12 3D HR (HP Inc., Palo Alto, CA, USA) with a 3D printer HP Jet Fusion 3D 4200 (HP Inc., Palo Alto, CA, USA). Following the printing process, the cutting guides underwent sandblasting as part of the post-processing procedure.

#### 2.4.2. Conventional Osteotomy (Models 1–5)

**Figure 3:**
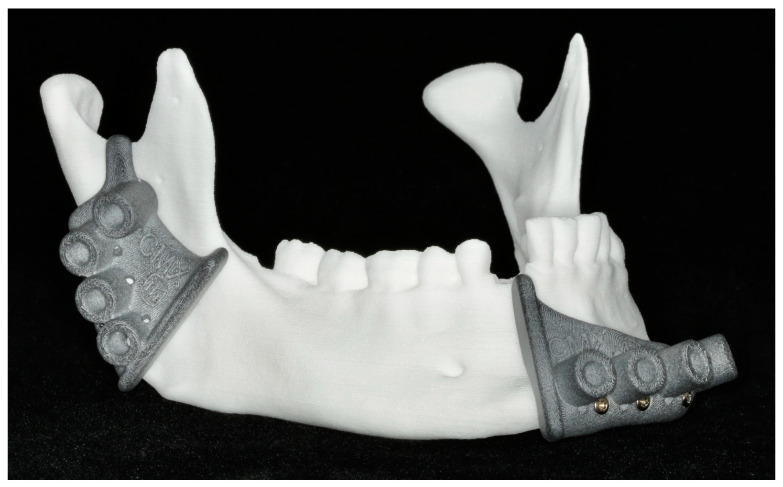
Mandibular model after placement of the cutting guides.

We performed conventional osteotomies on Models 1–5 while fixing cutting guides to the models with 2.0 mm-diameter cortical screws (Medartis AG, Basel, Switzerland) prior to the segmentectomy of the mandible. We used the Colibri II (DePuy Synthes, Johnson & Johnson, West Chester, PA, USA) for the osteotomy and a Twist Drill 1.6 mm for the 2.0 mm-diameter bicortical screws (Medartis AG, Basel, Switzerland) to drill the screw holes for the patient-specific implant. Presented below is one mandibular model with fixed cutting guides (Figure 3).

### 2.5. Laser Osteotomy by Robot Guidance

#### 2.5.1. Osteotomy Design

We selected the STL-Planning of Model 1 to devise osteotomy lines for the laser segmentectomy and to determine the locations of drill holes for subsequent osteosynthesis. Utilizing Geomagic Freeform (3D Systems Inc., Rock Hill, SC, USA), a patient-specific reconstruction plate was planned based on the osteotomy design, incorporating the positions of the drill holes. Then, we transferred the planning data to the CARLO^®^ primo+ software v. 2.0.x (AOT AG, Basel, Switzerland).

#### 2.5.2. Laser Osteotomy (Models 6–10)

**Figure 4:**
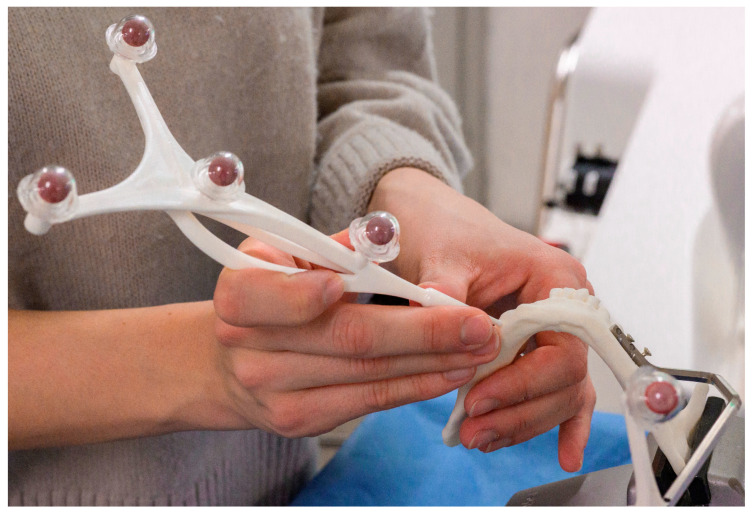
Landmark registration with the pointer.

Firstly, we calibrated a patient marker and a round pointer. The patient marker, which is shown below (Figure 4), was firmly fixed with screws to the left corpus of every mandibular model. Next, each model underwent registration, wherein anatomical landmarks were identified on the touchscreen interface of the CARLO^®^. These landmarks were located and relayed to the navigation system using the round pointer. We defined the right mental foramen, the distal incisal edge of the lower second right incisor, the middle protuberance of the last right molar, and the distal protuberance of the second last left molar as landmarks. Then, we conducted an additional surface registration with the round pointer to improve the registration accuracy after the landmark registration. Subsequently, the mandatory registration check was completed.

**Figure 5:**
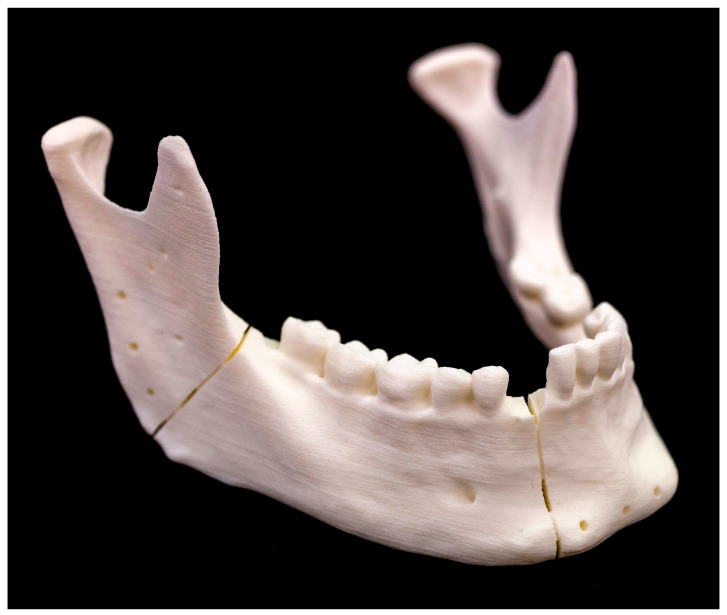
The 3D printed mandibular model after performing the osteotomy halfway and drilling the screw holes.

As we performed the laser osteotomy on Models 6–10, the presented sections exhibited clear edges with no indications of carbonization or charring. The reference points on the right ascending mandibular ramus are visible as small depressions. The laser process caused the initially planned 1.0 mm-diameter screw holes on the outer edge of the right ascending mandibular ramus and the frontal mandibular area to result in larger 1.7 mm-diameter holes due to material loss. The 1.7 mm diameter for the holes was chosen to ensure optimal initial mechanical stability for the 2.0 mm-diameter screws. Presented below is one mandibular model after osteotomy (Figure 5).

### 2.6. Patient-Specific Reconstruction

#### 2.6.1. Patient-Specific Implant Planning and Fabrication

The STL-Planning file also served as the basis for the virtual planning of a patient-specific implant in Geomagic Freeform (3D Systems Inc., Rock Hill, SC, USA). It is designed with a 0.3 mm offset from the bony mandibular surface and four screw holes on each end. Ten patient-specific implants were fabricated from a solid titanium blank, according to the planning data. After milling, the plates were post-processed in accordance with established standards by the implant manufacturer. Thus, the plates were milled according to the mandible’s anatomy to fit the digital preoperative plan and drill holes for osteosynthesis precisely. The plates had a thickness of 2.3 mm and a width of 8.0 mm.

#### 2.6.2. Reconstruction with Patient-Specific Implant

**Figure 6:**
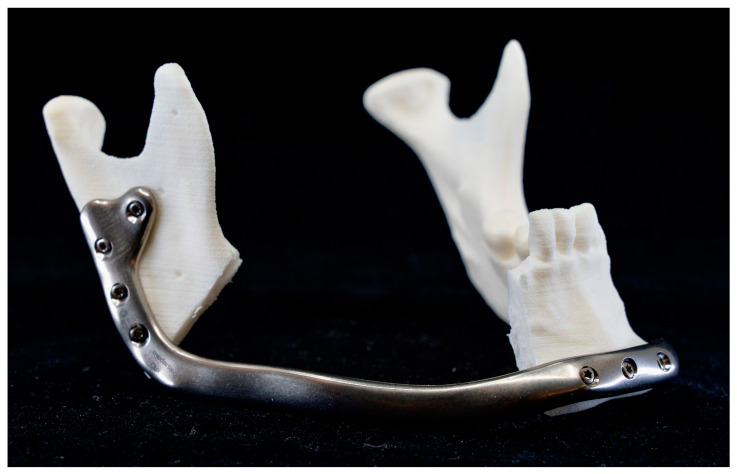
Mandibular model after reconstruction with a patient-specific implant.

We performed reconstruction manually on all ten models using the previously fabricated titanium patient-specific implants. These implants were fixed to the bone using a TriLock System (Medartis AG, Basel, Switzerland). Bicortical screws with a length between 7 and 15 mm as well as a 2.0 mm diameter were applied. A reconstructed mandibular model is shown below (Figure 6).

#### 2.6.3. Digitalization Process 3

Following the reconstruction process applied to the ten mandibular models, each replica underwent rescanning using the identical pre-established procedure. This process yielded an additional set of ten STL files labeled as STL-Reconstruction 1–10.

### 2.7. Accuracy Assessment

After registration, the ten reconstructed mandibles were compared with the reference model and each other. More precisely, the ten STL-Reconstruction files were compared with the STL-Planning file of Model 1. This allowed us to determine the accuracy regarding the trueness of the reconstruction (Comparison 1: n = 10). The STL-Reconstruction files 1–5 were compared with each other, as were the STL-Reconstruction files 6–10, to assess the accuracy regarding precision. On the one hand, a comparison was made within the conventional osteotomy group (comparison 2: n = 10) and, on the other hand, within the laser osteotomy group (comparison 3: n = 10). Then, the laser osteotomy was compared with the conventional osteotomy group (comparison 4: n = 25). The 3D analysis software 3-matic medical v. 17.0 (Materialise, NV, Leuven, Belgium) was used for the assessment. The overlay of models involved reference points located on the right and left ascending mandibular ramus and an additional reference point on the front teeth. The superimposition of the models entailed two alignments with an n-point registration and with the manually positioned reference points. After overlaying the parts, a part comparison analysis was conducted, and a heatmap was generated to illustrate the areas of aberrance.

### 2.8. Evaluation

In this context, trueness refers to the degree to which the arithmetic mean of the test findings corresponds with the true or accepted reference value (paragraphs 0.2 and 0.4, ISO 5725-1) [29]. Therefore, trueness was assessed by comparing the reconstructed mandibular models with the STL-Planning file of Model 1. Precision is defined as the degree of agreement between the results of independent tests performed under specified conditions (paragraphs 0.2 and 0.4, ISO 5725-1) [29]. It refers to the statistical or random mistakes resulting from repeated measurements. Hence, it also refers to the variability in repeatedly taken measurements [30]. Precision was assessed by comparing the individual osteotomy technologies within their respective groups.

### 2.9. Statistics

Statistical analyses assessed trueness by comparing the reconstructed mandibular models to the reference data and determining the closest results. Additionally, the analyses assessed precision by evaluating the consistency of the results across the different reconstructions performed using the appropriate osteotomy technique. Descriptive data analysis summarized the mean, standard deviation (SD), median, minimum, maximum, and root mean square (RMS) values of the surface comparison of the 3D printed models using both osteotomy technologies. Then, the Shapiro–Wilk test and QQ plots were used to assess the distribution of the RMS values for trueness and precision. Given that most distributions deviated from normality, the Wilcoxon rank sum test was used to compare the RMS values between robot-guided laser osteotomies and conventional osteotomies. All statistical analyses were performed using the R statistical software (v. 4.2.2, The R Foundation for Statistical Computing, Vienna, Austria).

## 3. Results

The accuracy, made up of precision and trueness, was analyzed based on the superimpositions of the scanned reconstructed mandibles on the reference STL-Planning file. The following sections characterize and illustrate these accuracy terms.

### 3.1. Trueness

**Figure 7:**
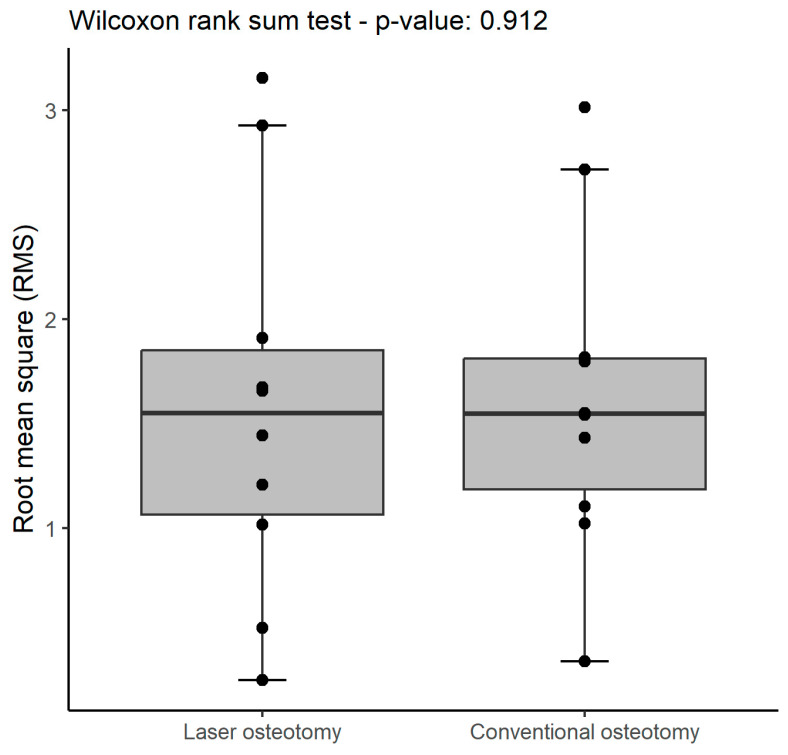
Box plot demonstrating trueness RMS values (mm) by osteotomy technology.

A summary of the statistics for trueness is shown below (Table 1). No statistically significant difference in trueness RMS values was observed (Table 2). Overall, the conventional osteotomy group exhibited higher values compared to the laser osteotomy group, except for one maximum value. This is also illustrated in the box plot (Figure 7), where the laser osteotomy group shows greater variability compared to the generally lower values of the conventional osteotomy group.

**Figure 8:**
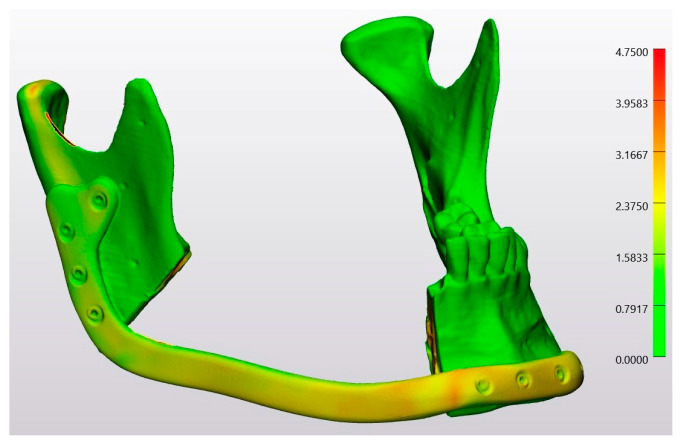
Heatmap of the superimposition of Model 4 on the STL-Planning file.

**Figure 9:**
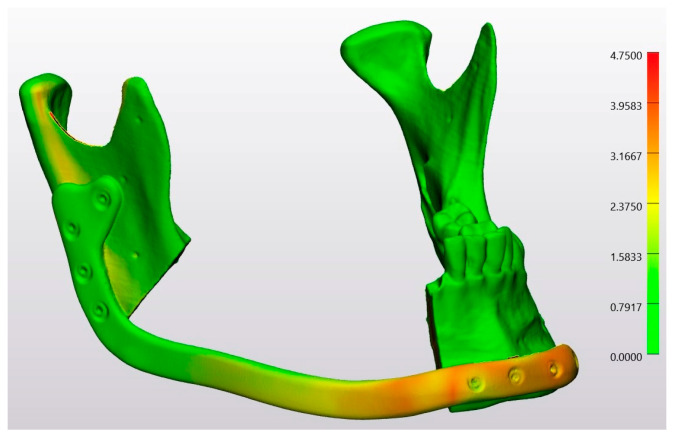
Heatmap of the superimposition of Model 6 on the STL-Planning file.

In a heatmap, the conventional osteotomy group was compared with the planning file by superimposing Model 4 on the STL-Planning file (Figure 8). Similarly, the laser osteotomy group was compared to the planning file by superimposing Model 6 on the STL-Planning file (Figure 9). Since the analysis was conducted with unsigned values, all deviations are represented as positive. Red spots denote positive deviations, while green indicates a high degree of agreement between the values and, thus, minimal divergence from the comparison object.

### 3.2. Precision

**Figure 10:**
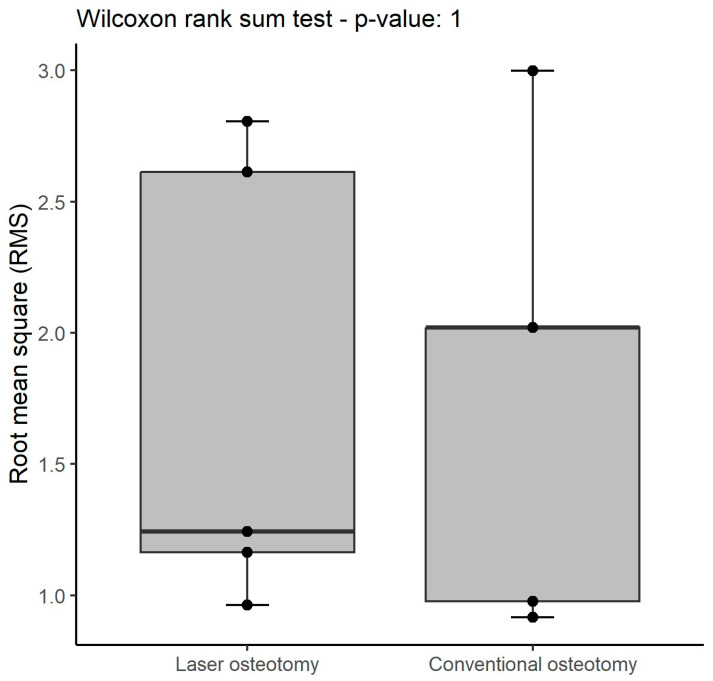
Box plot demonstrating precision RMS values (mm) by osteotomy technology.

There was no statistically significant difference observed in precision RMS values between the two technologies. While the study might have lacked sufficient power to detect minor differences in precision, the values appeared remarkably comparable across all groups. A comprehensive summary of all precision statistics is presented below (Table 3). The RMS values pertaining to precision are identical for both groups (Table 4). These values exhibit overall similarity, except for the maximum value, which once again presents higher values in the laser osteotomy group. Nevertheless, the conventional osteotomy’s values display less dispersion (Figure 10).

**Figure 11:**
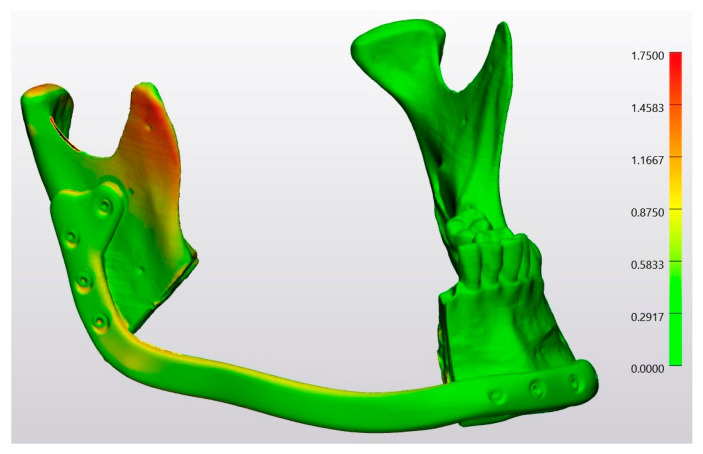
Heatmap of the superimposition of Model 3 on Model 4.

**Figure 12:**
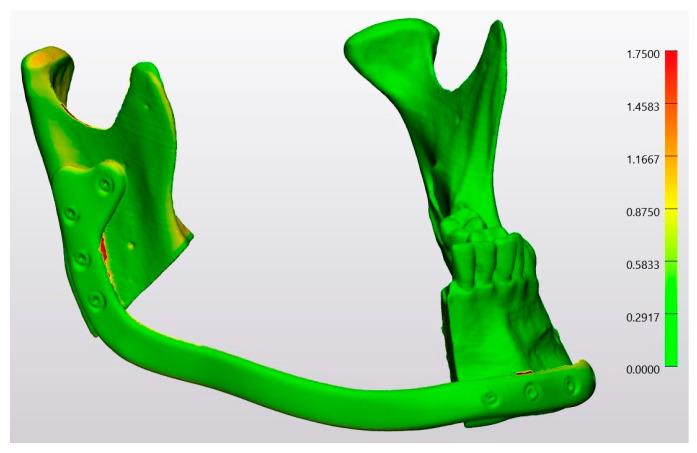
Heatmap of the superimposition of Model 8 on Model 9.

In a heatmap, the conventional osteotomy group was compared by superimposing Model 3 on Model 4 (Figure 11). Similarly, the laser osteotomy group was compared by superimposing Model 8 on Model 9 (Figure 12). The green color accounted for most of the models.

## 4. Discussion

Inaccurate preoperative planning poses risks such as compromised outcomes, potential complications, or even patient harm. The foremost objective of any reconstructive procedure is to restore the original anatomical state as accurately as possible. Achieving this objective entails maintaining established workflows and ensuring consistent clinical outcomes. Newly developed technologies for reconstruction undergo constant testing and modification to accomplish this [11,31]. This study compared a conventional guide-dependent osteotomy, the accepted norm for osteotomies, with a robot-guided laser osteotomy in terms of accuracy.

Accuracy constitutes a foundational aspect of reconstructive surgery. In the present study, the root mean square was used to evaluate trueness and precision, key components in assessing accuracy according to ISO 5725-1 [29]. Accuracy is typically described using a standard deviation. However, alternative methods for representing data variability, such as the root mean square, can also be employed. Using the root mean square offers distinct advantages, as the values may more accurately reflect the measured data, unlike the mean, which can be influenced by individual extreme values that may cancel each other out [30,32]. The deviation between a virtual planning file and the scan of a reconstructed mandibular model was assessed by superimposing two STL files. Divergence can be bidirectional, resulting in positive and negative (signed) values. However, minimum and maximum values, representing the absolute distance from the origin rather than a direction, were utilized as unsigned values. The objective was to assess the extent to which our reconstructions deviated from the target.

Advancements in medical and technical development have prompted efforts to simplify surgical procedures and improve their outcomes. Numerous studies, including those of Mazzoni et al., have demonstrated the successful and effective integration of preoperative surgical planning and cutting guides [16]. Adopting entirely digital preoperative planning offers significant benefits in terms of visualization and time-saving measures during surgery. When integrated with a digital workflow and robot-guided surgery, it not only yields substantial savings in time and cost but also enhances intraoperative safety. By shifting the time-consuming stages to the preoperative phase or eliminating them altogether, additional time efficiencies are gained, such as by eliminating the necessity for cutting guide planning and printing [2,12]. Incorporating robotic technology in the operating room has been shown to integrate seamlessly into current surgical workflows with minimal disruption [33].

Although cutting guides were introduced to simplify the intraoperative workflow and enhance precision, they incur additional costs and time for planning, production, and positioning, including fixation with additional screws. However, shifting these time-consuming tasks to the preoperative phase still offers the advantage of reducing overall surgery time [34,35]. Accurately placing the cutting guides can be challenging due to complexities in identifying the virtually planned position or fitting inaccuracies. It is essential to produce a cutting guide with high precision. However, in a clinical setting, challenges may arise during positioning due to soft tissue residues on or around the bone, fabrication inaccuracies such as shrinkage, or errors in the planning process. Therefore, creating positioning aids requires balancing the need for a perfect anatomical fit and positioning while ensuring optimal functionality [34,36]. Furthermore, positioning the guides often necessitates greater bone exposure, leading to increased soft tissue trauma. Guides may also obstruct the bone surface, impeding effective cooling. Moreover, the space in the guiding holes within the printed cutting guides, designed for drill insertion, poses an additional challenge. While this space accommodates the supplementary drill guide piston, it allows for minimal angulation or offset during screw positioning. This design feature could complicate the fixing of the osteosynthesis plate, potentially causing a misalignment between the screw positions on the plate and the screw holes in the bone.

Previous studies have introduced workflows for implant fabrication to facilitate facial reconstruction, considering feasibility, cost-effectiveness, force resistance, and accuracy [27,32,37,38]. While patient-specific implants provide significant time-saving benefits during surgery by eliminating the need for manual adjustments such as bending, it is essential to consider the potential for inaccuracies in their fabrication. Typically, milled titanium plates are preferred for patient-specific reconstruction. Milling, a subtractive fabrication process, involves carving a plate out of a solid titanium block. This approach allows for preoperative planning and patient-specific customization and ensures robust mechanical properties. Such plates are especially suitable for reconstructing significant defects, as they sidestep the need for intraoperative adjustments and maintain their mechanical stability and structural integrity. However, certain drawbacks are associated with the production of titanium plates through milling, including substantial material loss and increased cost. Although these plates are customized to fit a patient’s unique anatomy, certain anatomical complexities may remain inaccessible. Additional adjustments, such as smoothing and leveling, and further considerations in the planning process are required to accommodate solid overhangs or uneven surfaces. In cases where incorporating bone relief into the plate is crucial, 3D printing may offer advantages [31]. Compared to milled plates, 3D printed titanium plates may exhibit reduced stability. However, their closer proximity to the bone surface is an advantage in certain cases. On the other hand, milled implants balance stability and patient-specific treatment as well as save time during surgical procedures [39].

The challenges associated with accurately positioning the 3D printed cutting guides underscore the advantages of a robot-guided system. Numerous sources of inaccuracy can be mitigated by faithfully executing the preoperatively planned cutting path. Using a robot-guided laser osteotome, the cutting path is visually displayed beforehand with a green laser. This not only enhances safety and accuracy but also allows for the flexibility to make adjustments intraoperatively when necessary. The ability to make real-time intraoperative adjustments to the cutting plan represents a significant improvement over conventional osteotomy methods, offering valuable benefits in terms of surgical accuracy and adaptability [2]. Even though the digital workflow saves time, executing a robot-guided osteotomy requires additional effort. The robotic arm must be calibrated and positioned precisely before the bone can be cut efficiently, which involves the use of a patient marker and a pointer. In the literature, several studies have investigated the feasibility of the CARLO^®^ and other robot-guided laser systems, collectively suggesting a commendable level of accuracy in these techniques [2,7,12,15,18,19,23,38,40,41]. The existing literature indicates that the combination of a robot-guided laser system with a navigation system offers a number of advantages in the performance of precise osteotomies with freely selectable geometries [14]. Furthermore, the technical innovation has demonstrated considerable promise in terms of accuracy and safety in the context of orthognathic surgery [12]. A further study on cadavers has proven the accuracy of bone channels for the implantation of depth electrodes in epilepsy surgery using this technology [41]. Some studies indicate that robot-guided surgical systems have the potential to replace certain manually performed techniques and mitigate the risks associated with human errors [39]. Moreover, recent literature has explored novel applications and prospects for robot-guided laser technology. For instance, Honigmann et al. conducted a study assessing the potential use of robot-guided laser osteotomies in hand, wrist, and forearm surgery on human cadavers, shedding new light on these applications [42]. In a separate study, Wojcik et al. assessed the feasibility of complex unicortical calvarial harvesting through robot-guided laser osteotomy, expanding the horizons of this technology [40]. However, a literature review has revealed limited information on robot-guided surgical systems for osteotomies and subsequent reconstruction, which implies the necessity for more research in this field. While current studies have delved into the accuracy of digital workflows, comprehensive research in this domain remains scarce [11,43]. For instance, Chao et al. conducted a robot-guided osteotomy on printed fibula models, focusing on osteotomy accuracy, yet reconstruction was not included in their analysis [43]. Most of the studies have primarily focused on the feasibility and accuracy of the osteotomy procedure itself, often without addressing the reconstruction phase. From a clinical perspective, performing an osteotomy with an offset of a small fraction has an insignificant impact on the final reconstruction outcome. Nevertheless, precise alignment between the osteotomy line and drilled screw holes is crucial, as any misalignment would lead to significant deviations in accuracy and improper fitting of the osteosynthesis plate. Therefore, it is essential to include reconstruction as an endpoint in research studies, as was implemented in this one.

As mentioned previously, numerous studies have also emphasized the positive biological impacts of laser-assisted bone cutting [1,15,17,24,26,27,39,44,45]. Distinct dissimilarities are evident when looking at the heat development in both processes. Unlike conventional osteotomies, which cause the polyamide material to partially melt during cutting, laser osteotomies do not produce this effect. These studies collectively show that robot-guided laser osteotomy offers several advantages, including improved accuracy and safety. Moreover, after considering the manifold benefits of laser technology, performing osteotomies with this cutting-edge method presents itself as a robust surgical technique with significant potential advantages.

It should be noted that the current study is not without limitations. The sample size for both osteotomy groups is relatively small. Given that the robot is still in the developmental phase, the financial costs are still considerable, with significant labor inputs required in terms of registration and material costs. However, as surgeons become more familiar with robotic technology, the time and resources required for preoperative planning and robotic programming will likely decrease. Larger sample sizes would have been preferable to enable more comprehensive comparisons and establish statistically significant differences. Nevertheless, it is important to note that the results from the two osteotomy groups displayed striking similarities, with neither method demonstrating clear superiority. Another limitation of this study was the application of a pointer with a rather imprecise round tip. In our study, the pointer’s tip was rounded; however, greater accuracy could be achieved using a sharper tip. As this could be the cause of certain inaccuracies, a sharp tip or even a surface scan could have been beneficial. To further enhance registration accuracy and minimize errors, an optical surface scanner is currently under development. This surface scanner offers the advantage of registering an entire point cloud rather than individual points [46]. However, the software provided sufficient values after the registration check without displaying any errors. It is also important to consider certain technical factors, such as the osteotomy path. The preoperative virtual planning path determines the osteotomy line, but it does not yet determine the osteotomy depth. Currently, optical systems are being investigated that analyze the osteotomy depth. Other potential technical errors may arise during the transfer of planning to another software, the robot’s registration process, the laser’s osteotomy performance, and the production and fixation of the plate. The presence or absence of soft tissue during surgical procedures involving bones becomes a critical factor for the repositioning maneuver. The soft tissue was absent in our study, creating an environment that allowed unhindered positioning and manipulation during osteotomy and subsequent reconstruction. We faced an artificial scenario in mandibular reconstruction, as this unique condition enabled us to freely maneuver the two mandible parts after the osteotomy and securely join them by hand. Finally, a 3D printed reconstruction plate could provide a more accurate surface fit than milled plates, which is a potential improvement. However, it needs to be mentioned that such a printed plate differs from the typical option available on the market, which usually comprises milled plates.

This study highlights the numerous advantages of a robot-guided laser osteotome, as underscored by existing research, particularly in terms of accurate surgical procedures. Robotic surgery offers several benefits, including more precise access to the surgical site, smaller scars, and fewer post-operative complications. Advances in robotic technology have led to improved features such as 3D imaging, touchscreen displays, real-time navigation, and haptic feedback. In the future, such a system will not only be able to cut, but will also be able to combine a wide range of procedures and benefits, from the biological aspects of a laser to intraoperative bone repositioning and replacement of the surgical assistant.

Nevertheless, there remains a clear need for further investigations, including those exploring clinical outcomes such as reconstruction accuracy. This study provides important insights into acquiring additional knowledge that can be implemented in clinical settings and shaping the future of this innovative technology. Continued investigation and exploration with forthcoming research are essential for unraveling the complexities associated with an entirely digital workflow, a robot-guided laser osteotome, and the assimilation of patient-specific implants.

## 5. Conclusions

This new digital high-tech procedure proved to be equivalent to the conventional guide-dependent osteotomy. The conventional method, which includes cutting guides with drilling holes for subsequent reconstruction, demonstrated a median trueness RMS value of 2.0 mm (SD ± 1.7) and a precision of 1.6 mm (SD ± 1.4). The robot-guided laser osteotomy group had a median trueness RMS value of 1.2 mm (SD ± 1.1) and a precision of 1.6 mm (SD ± 1.4). Despite the small sample size, the two techniques show a similar accuracy after reconstruction. In order to overcome the current limitations, it would be beneficial to increase the sample size, thereby reducing statistical noise and potentially obtaining more reliable results.

The combination of a robotic arm with a navigation system and a laser head enables the accurate implementation of virtual planning and utilizes the biological aspects of a laser osteotome. It closes the gap between virtual planning on a workstation and the exact execution within the human body. This high-tech procedure is still in its early stages and has plenty of potential for adjustments and modifications; nevertheless, more studies are essential to underline these benefits.

## Figures and Tables

**Table 1:** Summary of all trueness values (mm) by osteotomy technology.

Technology	RMS	Mean	SD	Median	Minimum	Maximum
Laser Osteotomy	1.2	0.9	1.1	0.1	0.0	15
Conventional Osteotomy	2.0	0.9	1.7	0.1	0.0	12

**Table 2:** Comparison of all trueness RMS values (mm) by osteotomy technology.

	Laser Osteotomy	Conventional Osteotomy	
	Median (IQR)	Median (IQR)	*p*-Value
RMS (all)	1.2 (1.2 to 2.6)	2.0 (1.0 to 2.0)	1

**Table 3:** Summary of all precision values (mm) by osteotomy technology.

Technology	RMS	Mean	SD	Median	Minimum	Maximum
Laser Osteotomy	1.6	0.7	1.4	0.1	0.0	17
Conventional Osteotomy	1.6	0.8	1.4	0.1	0.0	15

**Table 4:** Comparison of the precision RMS values (mm) by osteotomy technology.

	Laser Osteotomy	Conventional Osteotomy	*p*-Value
	Median (IQR)	Median (IQR)	
RMS (all)	1.6 (1.1 to 1.9)	1.6 (1.2 to 1.8)	0.9

## Abbreviations

3D	three-dimensional
CARLO	cold ablation robot-guided laser osteotome
CO_2_	carbon dioxide
CT	computed tomography
Er:YAG	Erbium-doped Yttrium Aluminum Garnet
IQR	interquartile range
ISO	International Organization for Standardization
Nd:YAG	Neodymium-doped Yttrium Aluminum Garnet
QQ	quantile–quantile
RMS	root mean square
SD	standard deviation
SLS	selective laser sintering
STL	standard tessellation language
